# TGF-β1 induces HMGA1 expression in human breast cancer cells: Implications of the involvement of HMGA1 in TGF-β signaling

**DOI:** 10.3892/ijmm.2015.2062

**Published:** 2015-01-05

**Authors:** XUYU ZU, JING ZHONG, JINGJING TAN, LI TAN, DONG YANG, QINGHAI ZHANG, WENJUN DING, WEN LIU, GEBO WEN, JIANGHUA LIU, RENXIAN CAO, YUYANG JIANG

**Affiliations:** 1 Institute of Clinical Medicine, The First Affiliated Hospital of University of South China, Hengyang, Hunan 421001, P.R. China; 2 Guangdong Provincial Key Laboratory of Chemical Biology, Graduate School at Shenzhen, Tsinghua University, Shenzhen, Guangdong 518055, P.R. China

**Keywords:** high mobility group A1, transforming growth factor-β1, breast cancer, human epidermal growth factor receptor 2

## Abstract

Transforming growth factor-β1 (TGF-β1) signaling and high mobility group A (HMGA1) are known to play essential roles in the progression of breast cancer by inducing epithelial-mesenchymal transition. However, the correlation between TGF-β1 and HMGA1 in breast cancer cell is not yet well understood. In this study, we determined the effects of TGF-β1 on HMGA1 expression in breast cancer cells and examined the role of HMGA1 in breast cancer progression. Our results demonstrated that TGF-β1 induced the expression of HMGA1 in both MCF-7 and MDA-MB-231 breast cancer cells, as shown by RT-qPCR and immunofluorescence staining; however, the TGF-β1-induced expression of HMGA was blocked by treatment of the cells with phosphatidylinositol-3 kinase (PI3K) signaling inhibitors. Moreover, the HMGA1 promoter activity was found to be activated by TGF-β1 in the MCF-7 and MDA-MB-231 cells and we found that specificity protein 1 (Sp1) was involved in the TGF-β1-induced HMGA1 promoter activity, as shown by luciferase activity assay. Furthermore, the enforced expression of HMGA1 by transfection with a HMGA1 promoter enhanced cellular oncogenic properties, including proliferation, migration and invasion, and a tissue microarray revealed that breast tumors expressing human epidermal growth factor receptor 2 (HER2) showed higher expression levels of HMGA1 (P=0.007). In addition, higher HMGA1 expression levels were also observed in the ductal breast cancer cases compared with the lobular breast cancer cases (P=0.000). These findings establish the first link between HMGA1 and TGF-β1 in breast cancer, providing further evidence of the pivotal role of HMGA1 in breast cancer progression.

## Introduction

Breast cancer is one of the most malignant tumors occurring in females, and approximately 350,000 women worldwide succumb to the disease annually (1). A number of factors, including lifestyle, environmental, genetic and biological factors contribute to the initiation and progression of breast cancer. Transforming growth factor-β1 (TGF-β1) is secreted from breast cancer cells and plays an important role in the occurrence and development of breast cancer (2). TGF-β1 functions as a suppressor during the early phase of tumor progression and becomes a cancer-promoting modulator during the late stages of cancer. Classic TGF-β1 signaling functions through 2 types of serine/threonine kinase receptors. Upon ligand binding, the type II receptors activate the type I receptors, which activate and recruit Smad2 and Smad3 and activated Smad2 and Smad3 combined with Smad4 then translocate to the nucleus to regulate gene expression (3). Apart from the TGF-β1/Smad pathway, TGF-β1 can also carry out its role in tumor progression by activating non-Smad pathways, such as the phosphatidylinositol-3 kinase (PI3K), extracellular signal-regulated kinase [ERK, mitogen-activated protein kinase (MAPK)], c-Jun NH2-terminal kinase (JNK) and p38 MAPK pathways and Rho GTPases (4). TGF-β1 is commonly recognized as an essential promoter of epithelial-mesenchymal transition (EMT) and TGF-β1-induced EMT is considered to be an important initiator of the invasive behavior of tumors during cancer progression.

The high-mobility group A (HMGA) family has 3 proteins: HMGA1a, HMGA1b and HMGA2. Although these proteins do not show direct transcriptional regulation activity, they regulate the transcriptional activity of several genes by altering the chromatin structure (5–7). HMGA1 has been confirmed to exert an oncogenic effect on the initiation and progression of diverse types of tumors (6,8–14). The overexpression of HMGA1 has been observed in several malignant neoplasias, such as thyroid cancer, colon cancer, breast cancer, lung cancer, ovarian cancer and prostate carcinoma, as well as head and neck tumors (15–23). HMGA1 has also been reported to promote the progression of breast tumors by inducing EMT (24).

In this study, we aimed to determine the effects of TGF-β1 on the expression of HMGA1 in breast cancer cells. To the best of our knowledge, the present study provides the first link between TGF-β1 and HMGA1 in breast cancer cells and PI3K signaling and specificity protein 1 (Sp1) were found to be involved in the TGF-β1-induced expression of HMGA1. This study also offers further evidence of the pivotal role of HMGA1 in breast cancer progression.

## Materials and methods

### Cell culture, transfection and antibodies

Human MCF-7 and MDA-MB-231 breast cancer cells (American Type Culture Collection, Manassas, VA, USA) were cultured in 37°C in a humidified atmosphere containing 5% CO_2_. The cells were maintained in Dulbecco’s modified Eagle’s medium (DMEM) supplemented with 10% FBS (Gibco Life Technologies Australia Pty Ltd., Mulgrave, Victoria, Australia). The cells were transiently transfected with the PGL4/HMGA1 plasmid and normalized with the use of a co-transfected PTK construct using Lipofectamine 2000 (Invitrogen, Carlsbad, CA, USA). At 24 h after transfection, the cells were stimulated with the indicated concentrations of TGF-β1 for 12 h. A luciferase assay kit (Promega, Madison, WI, USA) was used to measure the reporter activity according to the manufacturer’s instructions. Luciferase activity was normalized using a *Renilla* luciferase internal control. The antibodies used for immunofluorescence and electrophoretic mobility shift assay (EMSA) were as follows: anti-HMGA1 (ab129153), anti-Sp1 (ab13370) and anti-p-Sp1 (ab59257) antibodies (Abcam, Cambridge, MA, USA).

### Plasmid construction

The HMGA1 promoter was subcloned into the pGL4.10 basic plasmid (Promega) at the *Kpn*I and *Hin*dIII restriction enzyme sites to drive luciferase expression. The primers used for amplification were 5′-GACGGTACCT GCTGGAGGCTGAGGAATCG-3′ and 5′-CAGAAGCTTTA GCAAATGCG GATCTGAAACC-3′ for a 2,164 bp fragment.

### RNA isolation and reverse transcription quantitative (real-time) PCR (RT-qPCR)

The MCF-7 cells and MDA-MB-231 cells were treated with or without LY294002 and wortmannin (Sigma, St. Louis, MO, USA), two selective inhibitors of PI3K for 2 h, and maintained in culture medium with TGF-β1 for 9 h. Total RNA was extracted from the MCF-7 cells or MDA-MB-231 cells using TRIzol reagent (Sigma) and the total RNA was reverse transcribed into cDNA using the first-strand synthesis kit (Gibco-BRL, Carlsbad, CA, USA). HMGA1 mRNA was amplified using the primers forward, 5′-AGGGAAGATGAGTGAGTCG-3′ and reverse, 5′-AAGCTG CTCCTCCAGTGAG-3′ and β-actin was utilized as a control using the primers forward, 5′-ATCTGGCACCACACCT-3′ and reverse, 5′-CGTCATACTCCTGCTT-3′. Quantitative PCR was performed using iQ SYBR-Green Supermix and regulated using a spectrofluorimetric thermal iCycler iQ5 (Bio-Rad, Hercules, CA, USA). The gene-specific primers were amplified with a denaturation step (95°C for 3 min), followed by 39 cycles of denaturation (95°C for 10 sec), annealing (55°C for 10 sec) and extension (72°C for 30 sec). Quantitative values were obtained from the cycle threshold (Ct) value. Samples from 3 separate experiments were analyzed in duplicate. The results from RT-qPCR were expressed as 2^-ΔΔCT^ using β-actin as a reference.

### Immunofluorescence

The cells were seeded on a round glass cover placed into a 6 well microtiter plate (Corning Life Sciences, Oneonta, NY, USA). The cells treated with 10 ng/ml TGF-β1 (Sigma) were fixed with 4% paraformal-dehyde-PBS for 15 min at room temperature, washed with PBS twice and and then permeabilized by incubation with 0.1% Triton X-100 for 5 min. The cells were washed twice with PBS again and stained with anti-HMGA1 antibody for 2 h at room temperature. After washing with PBS, each sample was incubated with Alexa Fluor-conjugated secondary antibodies (Boster, Wuhan, China) for 1 h. The samples were analyzed under a fluorescence microscope (Olympus, Tokyo, Japan).

### Luciferase report gene assay

The cells were cultured in serum-free medium for 12 h in 12-well plates. The cells were then transfected with 0.5 *μ*g of the HMGA1 vector containing luciferase or with the empty control vector, PGL4.1, using Lipofectamine 2000 (Invitrogen). After 6 h of transfection, the cells were treated with or without TGF-β1 (Sigma) at the indicated concentrations for addition 12 h. Luciferase activity was assessed using the Luciferase assay system (Promega) and was normalized using a *Renilla* luciferase internal control.

### EMSA

EMSA was performed using the LightShift Chemiluminescent EMSA kit (Pierce Biotechnology, Rockford, IL, USA) with slight modifications. Briefly, 5 *μ*g of nuclear extracts were pre-incubated with 50 ng-*μ*l poly(deoxyinosinic-deoxycytidylic acid), 10 mmol/l Tris-HCl (pH 7.5), 50 mmol/l KCl, 1 mmol/l dithiothreitol (DTT), 5 mmol/l MgCl_2_ and 0.05% NP-40 for 10 min at room temperature. Following pre- incubation, the samples were incubated for 10 min at room temperature with a biotin-labeled probe (5′-ATTCGATCGG GGCGGGGCGAGC-3′) or an unlabeled competitor probe (200-fold molar excess), or incubated for 60 min at 4°C with the appropriate antibody. Electrophoresis, electrophoretic transfer and the detection of the biotin-labeled DNA were carried out according to the manufacturer’s instructions.

### Cell proliferation, migration and invasion assays

For proliferation assays, cells were seeded (5,000 cells/well) and counted using an automated cell counter (Nexcelom Bioscience, Lawrence, MA, USA). For colony formation assay, cells were seeded (500 cells/well) and maintained for 7 days. Each experiment was carried out in triplicate and performed at least twice. For the invasion assays, 10,000 cells were resuspended in serum-free medium and placed in the upper chamber of a 24-well Matrigel™ Invasion Chamber (BD Biosciences, San Diego, CA, USA) coated with Matrigel. Cell invasion was calculated as the percentage of total cells that had invaded the bottom chamber containing complete medium with serum. Cell migration determined in a similar manner, except that Matrigel was omitted.

### Tissue microarray and immunohistochemical analysis

Tissue microarrays (BR1921a, BR10010a; US Biomax, Inc., Rockville, MD, USA), consisting 159 breast cancer cases and 32 normal cases and 50 paired cases of ductal breast cancer and corresponding lymph node metastasis with breast cancer, were utilized. The tissues were histologically interpretable and analyzed for the correlation with clinicopathological parameters. Immunohistochemical staining was performed as described in our previous study (25). Rabbit polyclonal HMGA1 antibody (1:50; Abcam) was used. Ethical approval for this study was obtained from the Human Research Ethics Advisory Committee of the University of South China, Hengyang, China.

### Statistical analysis

All experiments were performed with 3 replicates and the results were expressed as the means ± SEM. Statistical analysis was performed using SPSS software, version 13.0. χ^2^ tests were applied to assess the statistical significance. P-values <0.05 were considered to indicate statistically significant differences.

## Results

### Expression of HMGA1 is enhanced by TGF-β1 in breast cancer cells

**Figure 1:**
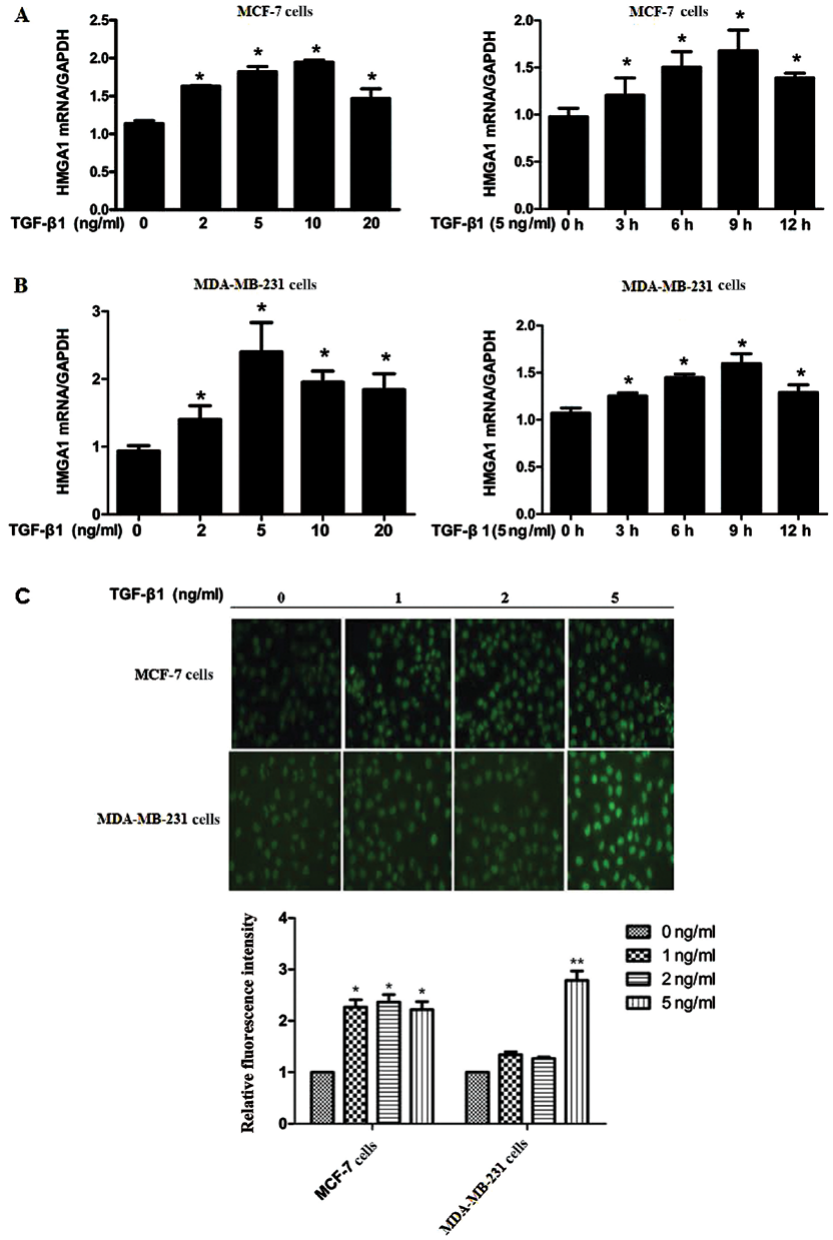
Transforming growth factor-β1 (TGF-β1) enhances high mobility group A1 (HMGA1) expression in MCF-7 and MDA-MB-231 cells. (A) qPCR analysis of HMGA1 expression induced by treatment with various concentrations of TGF-β1 for 9 h in MCF-7 and MDA-MB-231 cells. (B) qPCR analysis of HMGA1 expression induced by treatment with 5 ng/ml of TGF-β1 for the indicated periods of time. (C) Immunofluorescence staining of HMGA1 expression induced by treatment with various concentrations of TGF-β1 for 9 h in MCF-7 and MDA-MB-231 cells. ^*^P<0.05 and ^**^P<0.01, compared with the group not treated with TGF-β1.

TGF-β1 is an essential cytokine which has high expression level in the majority of mammary tumors, particularly in estrogen receptor (ER)-negative (ER^-^) tumors, stimulating mammary carcinoma cell invasion and metastatic potential (26). In this study, to investigate the effects of TGF-β1 on the expression of HMGA1, the 2 breast cancer cell lines, MCF-7 and MDA-MB-231, with or without ER expression, respectively were utilized. As shown in Fig. 1A, the HMGA1 mRNA level was increased by stimulation with TGF-β1 in both the MCF-7 and MDA-MB-231 cells in a dose- and time-dependent manner (Fig. 1A and B). Immunofluorescence assay revealed that the protein expression of HMGA1 was also elevated by TGF-β1 in both the MCF-7 and MDA-MB-231 cells (Fig. 1C). These data indicate that TGF-β1 functions as a positive modulator of HMGA1 expression in breast cancer cells independently of the existence of ER.

### TGF-β1 upregulates HMGA1 expression through the PI3K/Akt pathway

**Figure 2:**
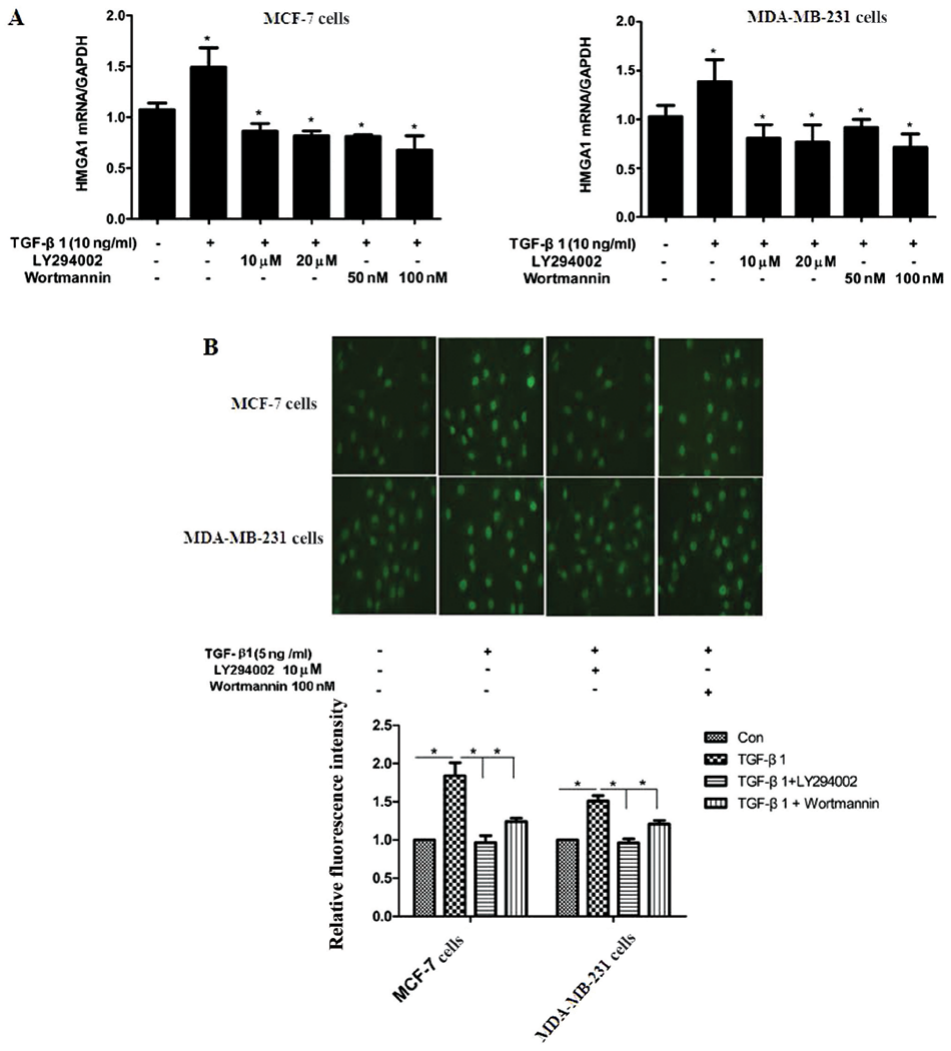
Transforming growth factor-β1 (TGF-β1) induces high mobility group A1 (HMGA1) expression through phosphatidylinositol-3 kinase (PI3K) signalling in human breast cancer cells. (A). qPCR was performed to assess the effects of LY294002 and wortmannin (inhibitors of PI3K) on the mRNA expression of HMGA1 induced by treatment with 10 ng/ml of TGF-β1 in MCF-7 and MBA-MD 231 cells. (B) Immunofluorescence staining of the effects of LY294002 and wortmannin on the expression of HMGA1 induced by treatment 5 ng-ml with TGF-β1in MCF-7 and MBA-MD-231 cells. ^*^P<0.05.

Several pathways and molecules, such as PI3K/Akt, MAPK and Smad3, have been shown to be involved in TGF-β1-induced cellular molecular events (27,28). LY294002 and wortmannin, 2 inhibitors of the PI3K/Akt pathway, were utilized to elucidate the underlying mechanisms of the TGF-β1-induced expression of HMGA1. As shown in Fig. 2A, the TGF-β1-induced mRNA expression of HMGA1 was abrogated by treatment with LY294002 and wortmannin in both the MCF-7 and MDA-MB-231 cells. Immunofluorescence staining also revealed that LY294002 and wortmannin reversed the TGF-β1-induced protein expression of HMGA1 in the MCF-7 and MDA-MB-231 cells (Fig. 2B). These results indicate the potential involvement of the PI3K/Akt pathway in the TGF-β1-induced expression of HMGA1 in breast cancer cells.

### TGF-β1 upregulates HMGA1 expression by enhancing the promoter activity of HMGA1 in breast cancer cells

**Figure 3:**
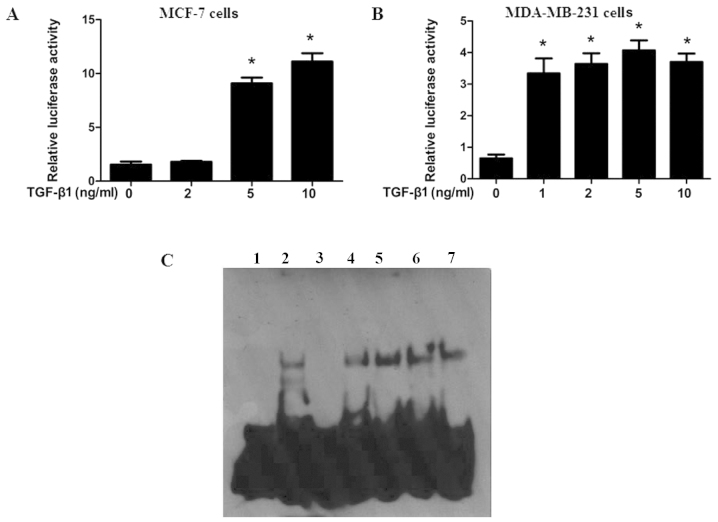
specificity protein 1 (Sp1) is involved in transforming growth factor-β1 (TGF-β1)-induced high mobility group A1 (HMGA1) promoter activity in breast cancer. (A and B) Effects of TGF-β1 on HMGA1 promoter activity. MCF-7 cells and MDA-MB-231 cells were transfected with the HMGA1 promoter/vector containing luciferase or with the empty control vector, PGL4.1, using Lipofectamine 2000 for 24 h, and the cells were treated with the indicated concentrations of TGF-β1. Twelve hours later, the cells were subjected to luciferase activity assay (n=3). (C) Binding of Sp1 and p-Sp1 (Thr453) to HMGA1 promoter was enriched following 12 h of TGF-β1 treatment in electrophoretic mobility shift assay (EMSA) (Materials and methods). Lane 1, probe; lane 2, probe + nucleic protein complex; lane 3, probe + nucleic protein complex + cold probe; lane 4, probe + nucleic protein complex + Sp1 antibody(−TGF-β1); lane 5, probe + nucleic protein complex + Sp1 antibody(+TGF-β1); lane 6, probe + nucleic protein complex +p-Sp1 antibody(-TGF-β1); lane 7, probe + nucleic protein complex + p-Sp1 antibody (+TGF-β1). ^*^P<0.01, compared with the group not treated with TGF-β1.

To further unravel the mechanisms through which TGF-β1 enhances the expression of HMGA1, the HMGA1 promoter sequence containing the GC box was obtained from the MCF-7 cells. As shown in Fig. 3A and B, TGF-β1 enhanced the promoter activity of HMGA1 in a dose-dependent manner in both the MCF-7 and MDA-MB-231 cells. Sp1 has been identified to be an essential transcription factor for the modulation of HMGA1 promoter activity by binding to the GC box located in the promoter sequence of HMGA1 (29). To further understand the role of Sp1 in the TGF-β1-induced expression of HMGA1, EMSA was performed on the breast cancer cells. It was found that TGF-β1 markedly increased the binding affinity of Sp1 to the HMGA1 promoter and the p-Sp1 binding to the HMGA1 promoter was also increased in response to TGF-β1 in the MCF-7 cells (Fig. 3C). These data suggest that Sp1 plays an essential role in the TGF-β1-induced expression of HMGA1 in breast cancer cells.

### HMGA1 enhances the proliferation and migration ability of breast cancer cells

**Figure 4:**
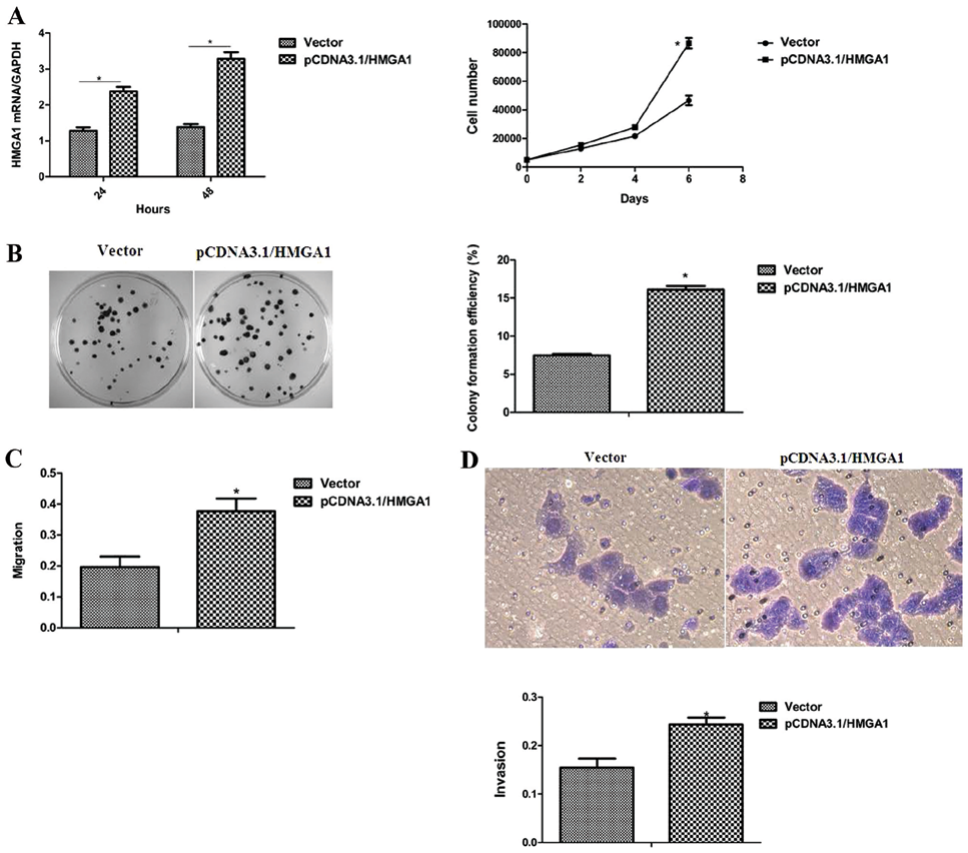
High mobility group A1 (HMGA1) promotes the oncogenic properties of breast cancer cells. (A) The ectopic expression of HMGA1 increased the proliferation ability of MCF-7 cells. (B) HMGA1 increased the colonic growth ability of MCF-7 cells. (C) HMGA1 enhanced the migration ability of MCF-7 cells. (D) HMGA1 enhanced the invasion ability of MCF-7 cells. ^*^P<0.05, compared with the vector control group.

To determine the role of HMGA1 in the oncogenic characteristics of breast cancer cells, the MCF-7 cells with an ectopic HMGA1 expression were utilized. As shown in Fig. 4A and B, the elevated expression of HMGA1 in the MCF-7 cells led to an obvious enhancement of the proliferation and colony-forming ability. The effects of HMGA1 on cell migration and invasion, properties of breast cancer progression, were further assessed and it was observed that the ectopic expression of HMGA1 in the MCF-7 cells markedly promoted cell migration and invasion (Fig. 4C and D). These results define an essential role of HMGA1 in the determination of the cellular oncogenic properties of breast cancer.

### The expression of HMGA1 is associated with the type of breast carcinoma

**Figure 5:**
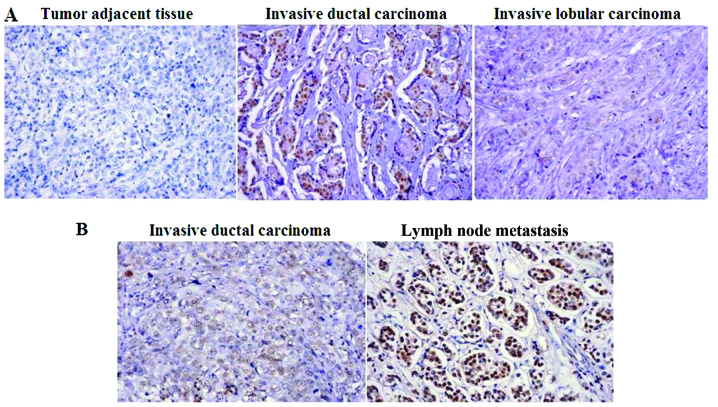
High mobility group A1 (HMGA1) expression is associated with the type of breast carcinoma (images in original magnification, x400). (A) Representative images of HMGA1 expression in tumor adjacent tissues, ductal and lobular breast tumor tissues. (B) Representative staining of HMGA1 in ductal breast tumor tissues and matched lymph node tissues.

To further indentify the association of HMGA1 with breast cancer progression, a tissue microarray (BR1921a; US Biomax, Inc.), consisting of 159 breast cancer cases and 32 breast tumor adjacent tissues was used. The tissue microarray analysis revealed that HMGA1 was expressed in 62.3 and 46.9% of the breast tumor tissues and matched breast cancer adjacent tissues, respectively, predominantly in the nucleus (Table I and Fig. 5A). Furthermore, statistical analysis confirmed that breast tumors with a high expression of human epidermal growth factor receptor 2 (HER2) showed a higher expression level of HMGA1 (P=0.007) and a higher expression level of HMGA1 was also found in the ductal breast cases compared with the lobular breast cancer cases (P=0.000) (Table II and Fig. 5A). These tissue microarray data suggest that a differential expression of HMGA1 exists in various types of breast cancer. To determine the association of HMGA1 with lymph node metastasis, a tissue microarray (BR10010a; US Biomax, Inc.), consisting of 50 paired samples of ductal breast cancer tissues and corresponding lymph node tissues, was utilized. It was found that 76% of the ductal breast cancer cases showed HMGA1 positive staining and 90% of the paired lymph node samples showed an expression of HMGA1 (Table III and Fig. 5B), although statistical analysis showed no significance (P=0.088).

## Discussion

HMGA1, as an oncogene, is enriched in adult stem cells and high-grade/poorly differentiated tumors (6,8–14) and it is highly expressed during embryogenesis with low or absent levels in adult tissues. HMGA1 is found to be overexpressed in almost all aggressive cancers and high levels of HMGA1 point to a poor prognosis in diverse types of tumors (18,20,21,30). HMGA1 functions in tumor progression by reprogramming differentiated cells into poorly differentiated, stem-like cancer cells. HMGA1 has also been demonstrated to be essential for the reprogramming of somatic cells to induce pluripotent stem cells (31).

TGF-β1 is a cytokine that modulates many fundamental aspects of cellular behavior, including growth, differentiation, migration and apoptosis. In early-stage adenomas, TGF-β1 functions as a tumor suppressor in normal epithelia by inhibiting cell growth and it is also one of the key cytokines in promoting EMT during embryonic development and during the late stages of cancer progression, leading to tumor cell invasiveness and metastasis. The present study provides evidence of a link between TGF-β1 and HMGA1 in breast cancer cells. It was found that TGF-β1 induced the expression of HMGA1 in both triple-positive breast cancer cells and triple-negative breast cancer cells. Since PI3K signaling has previously been described as one of the non-Smad signalings pathways which regulates TGF-β1 signaling pathway (4), we aimed to assess the involvement of PI3K signaling in the TGF-β1-induced HMGA1 expression. It was found that PI3K signaling was involved in this process; however, whether PI3K signaling operates in parallel or in direct coordination with the Smad proteins in the TGF-β1-induced epxression of HMGA1 remains to be further elucidated.

We further determined the downstream key molecules in the PI3K signaling pathway which mediate the TGF-β1- induced expression of HMGA1 in breast cancer cells. The effects of TGF-β1 on HMGA1 promoter activity were assessed and we found that TGF-β1 markedly increased the promoter activity of HMGA1 in both the MCF-7 and MD-321 cells. Sp1 has previously been confirmed to be an essential modulator of the regulation of HMGA1 promoter activity (29) and we determined the involvement of Sp1 in the TGF-β1-induced HMGA1 expression in breast cancer cells. It was revealed that TGF-β1 increased the binding affinity of Sp1 to the HMGA1 promoter *in vitro*, indicating that Sp1 takes part in the TGF-β1-induced expression of HMGA1. Sp1 phosporylation has been found to play an essential role in gene regulation (32). In a previous study, the phosporylation of Sp1 was shown to be involved in the gene promoter activity regulation of TRAIL, when vascular smooth muscle cells (VSMCs) were exposed to fibroblast growth factor (FGF)-2 (33). Our findings also suggest that TGF-β1 induces HMGA1 expression by activating Sp1 phosporylation, altering its occupancy at the HMGA1 promoter.

We also found that the ectopic expression of HMGA1 had a profound effect on oncogenic properties, including proliferation, migration and invasion and these results are consistent with those reported in the study by Shah *et al* (14) showing HMGA1 silencing in triple-negative breast cancer cells. To further unravel the significance of HMGA1 in clinical prognosis, we detected the expression of HMGA1 in a tissue microarray containing 159 breast cancer cases and 32 breast tumor adjacent tissues. We discovered that breast tumors with HER2 expression showed a higher expression level of HMGA1 and a higher expression level of HMGA1 was found in the ductal breast cancer cases compared with the lobular breast cancer cases. The associations of nuclear grade, tumor size and node metastasis with HMGA1 expression in breast tumor tissues was not found. Increasing evidence indicates that HMGA1 is of importance in maintaining a de-differentiated, pluripotent stem-like state (31) and HMGA1 has been demonstrated to reprogram somatic cells to induce pluripotent stem cells (iPSCs) (34). EMT is of importance for stem cells and metastatic tumors. The link between HMGA1 and EMT was revealed in MCF-7 cells, in which the enforced expression of HMGA1 resulted in metastatic progression and histological changes consistent with EMT (24). Although a growing body of evidences suggests the essential role of HMGA1 in tumor metastatic progression, we did not obtain the data for the correlation between HMGA1 expression and node metastasis in the tissue microarray. The disparity of HMGA1 between the cellular behaviors and clinical prognosis may be partly explained by the sample number used in this tissue microarray may not be sufficient and further investigations are required to clarify the importance of HMGA1 in the diagnosis and prognosis of breast cancer patients.

In conclusion, to the best of our knowledge, the present study provides the first evidence of the role of TGF-β1 in the regulation of HMGA1 expression in breast cancer cells and PI3K signaling and Sp1 were found to be involved in the TGF-β1-induced expression of HMGA1. Through Smads signaling, TGF-β1 is a well known inducer of EMT leading to tumor metastasis progression, and our data suggest that TGF-β1 promotes EMT by increasing the expression of HMGA1, offering a novel pathway for TGF-β1-induced EMT in breast cancer. Our study, together with data from previous studies, provides compelling evidence of the crucial role of HMGA1 in breast cancer progression.

## Figures and Tables

**Table I:** HMGA1 expression in breast carcinoma and breast cancer adjacent tissue.

Tissue type	n	HMGA1 (%)			P-value
		−	+	++	
Normal	32	17 (53.1)	13 (40.6)	2 (6.3)	0.102
Malignant	159	60 (37.7)	82 (51.6)	17 (10.7)	

HMGA1, high mobility group A1.

**Table II:** Correlation between HMGA1 expression and clinicopathological parameters.

Variables	n	HMGA1			P-value
		−	+	++	
Age (years), n (%)					0.077
<50	87	37 (42.5)	44 (50.6)	6 (6.9)	
≥50	72	23 (31.9)	38 (52.8)	11 (15.3)	
Tumor type, n (%)					0.000
Lobular	79	48 (60.8)	30 (38.0)	1 (1.3)	
Ductal	79	11 (13.9)	52 (65.8)	16 (20.3)	
Tumor size, n (%)					0.814
≤2 cm	18	6 (33.3)	12 (66.7)	0 (0.0)	
>2 cm	141	54 (38.3)	70 (49.6)	17 (12.1)	
Node metastasis, n (%)					0.208
Negative	93	39 (41.9)	45 (48.4)	9 (9.7)	
Positive	66	21 (31.8)	37 (56.1)	8 (12.1)	
Nuclear grade, n (%)					0.097
1	3	0 (0.0)	2 (66.7)	1 (33.3)	
2	60	6 (10.0)	40 (66.7)	14 (23.3)	
3	12	4 (33.3)	7 (58.3)	1 (8.3)	
ER, n (%)					0.090
Negative	104	43 (41.3)	53 (51.0)	8 (7.7)	
Positive	55	17 (30.9)	29 (52.7)	9 (16.4)	
PR, n (%)					0.105
Negative	116	46 (39.7)	62 (53.4)	8 (6.9)	
Positive	43	14 (32.6)	20 (46.5)	9 (20.9)	
HER2, n (%)					0.007
Negative	130	54 (41.5)	66 (50.8)	10 (7.7)	
Positive	29	6 (20.7)	16 (55.2)	7 (24.1)	

Data were from tissue microarrays (BR1921a), which consisted of 159 breast cancer cases, and were histologically interpretable and analyzed for the correlation with clinicopathological parameters. ER, estrogen receptor; PR, progesterone receptor; HER2, human epidermal growth factor receptor 2; HMGA1, high mobility group A1.

**Table III:** HMGA1 expression in breast carcinoma and breast cancer lymph node metastastic tissue.

Variables	n	HMGA1 (%)			P-value
		−	+	++	
Tissue type					0.088
Breast tumor	50	12 (24.0)	27 (54.0)	11 (22.0)	
Lymph node	50	5 (10.0)	33 (66.0)	12 (24.0)	

Data were from tissue microarrays (BR10010a), which consisted of 50 paired samples of breast cancer and breast cancer lymph node metastastic tissue. HMGA1, high mobility group A1.
